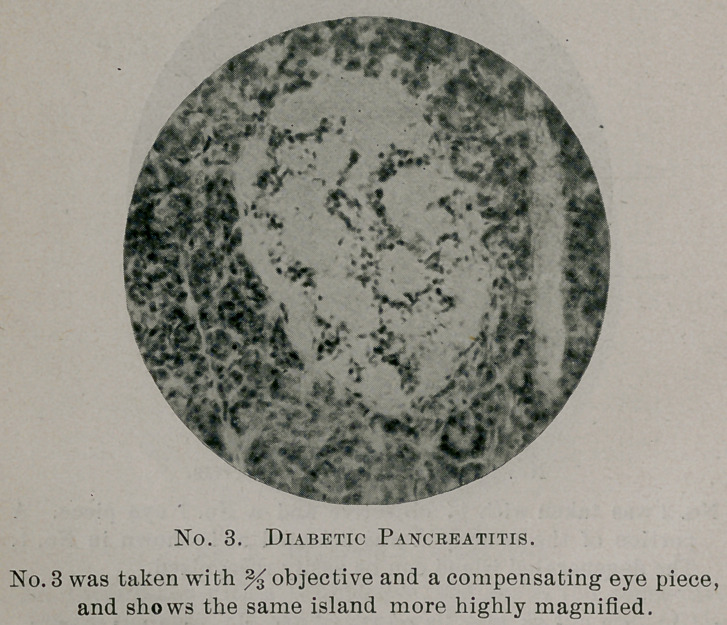# Post-Mortem Findings in a Case of Diabetes Mellitus

**Published:** 1904-03

**Authors:** Wm. C. Pumpelly

**Affiliations:** Pathologist State Sanitarium, Milledgeville, Ga.


### POST-MORTEM FINDINGS IN A CASE OF DIABETES MELLITUS.

By WM. C. PUMPELLY, M.D.,

Pathologist State Sanitarium, Milledgeville, Ga,

GEORGIA STATE SANITARIUM—AUTOPSY RECORD.

No. 258 ; Date, November 20, 1903 ; Name, Anthony Hodge ; Age, 60 years ; Occupation, farm-hand ; Hours after death, two (2); Race, Negro male ; Mental Disease, Senile Dementia ; Tuberculosis, no ; Brights, yes.

Urine Examination. August 28, 1903 ; Color, light amber ; Odor, peculiar ; Reaction, acid ; S/G, 1040 ; Sugar, 6.34 per cent.; Indican, a small amount ; Sediment, a small amount of flat-cell epithelium. (No casts or albumen found.)

Blood Examination. None made.

Clinical History. *Admitted*, August 24, 1904.

*Died*, November 20, 1903.

*Duration of mental disease*, 18 months.

*Examination on admission* (The history accompanying the commitment papers states : patient has had for about a year kidney and bladder disease with incontinence of urine, and it also states that the patient received an injury on the head some time before this).

Habits filthy, nutrition good, facial expression simple, patella reflex and ankle clonus absent. Radial and temporal arteries sclerosed, heart and lungs normal, liver extends slightly below the costal margin, skin is considerably bronzed.

He was very feeble when he came and his mental condition was almost a blank, and there was a gradual decline physically until his death. He had polyuria, incontinence of urine and bronzed skin and was in bed for a week before death.

Note. The incontinence of urine and the deranged mental condition seemed to be closely associated, as the history states that they were both noticed at the same time, about 18 months ago.

POST-MORTEM FINDINGS.

CASE 258. November 20, 1903, 1:30 P.M.

INSPECTION. Weight about 115 pounds, height 161 c.m. The body is emaciated and rigor-mortis is present.

HEAD. *Skull* is thin and the diploe are not well marked.

*Dura* is thickened.

*Pia-arachnoid* is opaque and edematous. The edema is most marked along the longitudinal sinus.

No. 1. DIABETIC PANCREATITIS.

No. 1 was taken with a 1/8 objective, and shows the connective tissue increase. It can be very well seen in the wall of the blood-vessel, and is also noticeable between the lobules.

*Brain*, weight 1100 gms. The brain is edematous and the convolutions are very much atrophied, the atrophy is most marked in the frontal region. The ventricles are full of fluid.

THORAX. *Lungs*, weight right 570 gms., left 605 gms. The right lung has a few adhesions along the anterior surface of the upper lobe and both lungs show well-marked hypostatic congestion at the base.

(Careful search was made for tubercular tissue but none was found.)

*Heart*, weight 230 gms. Is contracted and empty, the valves are normal.

ABDOMEN. *Liver*, weight 1200 gms. On section it appears congested. The lower edge of the left lobe is very thin.

*Kidney*, weight right 120 gms., left 170 gms. The capsule strips easily leaving a smooth surface, the cortex is thickened and the pyramids are well marked.

No. 2. DIABETIC PANCREATITIS.

No. 2 was taken with ⅙ objective and a No. 1 eye piece. A portion of the same field was selected as is shown in No. 1. The degenerated island can be easily recognized.

*Spleen*, weight 180 gms. The capsule is wrinkled and the consistency is increased.

*Colon* is packed full of hard fecal accumulations throughout its entire extent, from the cecum to the rectum.

MICROSCOPICAL EXAMINATION.

CORTEX. The larger vessels of the cortex are very sclerotic and the tortuosity of the smaller vessels is marked. The capillaries are here, as in all the other organs, over-distended with blood. They show increase of nuclei along their perivascular spaces, and both the perivascular and pericellular are everywhere greatly enlarged.

The nerve-cells are in an advanced stage of granular degeneration and small areas of softening can be occasionally seen along the outer surface of the cortex.

There is some round cell infiltration in the vicinity of the larger vessels and the glia cells in places seem to be increased both in size and numbers.

LUNGS. Sections taken from the base present the characteristic picture of hypostatic congestion; the alveoli are filled with blood and serum, and the septum is swollen and its capillaries congested.

NO. 3. DIABETIC PANCREATITIS.

No. 3 was taken with ⅔ objective and a compensating eye piece, and shows the same island more highly magnified.

LIVER. There is a slight amount of cloudy swelling throughout the liver, all of the blocks, which were taken from different portions of the organ, show it, and it is occasionally to be seen in advanced stages.

There are a few areas which take the hematoxylon stain diffusely, i.e., the protoplasm staining as well as the cell nuclei, and there are a few areas of hyaline degeneration. The parenchyma cells and the interspaces in many places are vacuolated, giving the appearance of fatty degeneration, but it is impossible to say whether they contained glycogen or fat, as both these substances are soluble in the fixing agents employed and were consequently dissolved out during that process in their preparation.

Spleen. The sections show marked congestion. The infiltration with blood-cells is extreme and is most marked in the medullary portion, where the spleen-cells are nearly masked by their presence. Sections stained with polychrom-methyl-blue show the red cells in the various stages of polychromophilic change. The connective tissue is increased to a moderate extent.

Kidney. The sections present a most typical picture of acute diffuse parenchymatous nephritis, the parenchyma cells are everywhere swollen and granular and the capillaries are distended with blood. There is a moderate amount of hyperplasia of the connective tissue and, in the proximal convolutions of Henle, there are some hyaline changes and a few casts are to be seen in the tubules.

Pancreas. There is some connective hyperplasia in all of the section taken; it is most noticeable in the capsule and trabecula and in the walls of the larger vessels and ducts. There is a perceptible increase of the interlobular tissue, though this is not uniform (this was ascertained by careful comparisons with sections of normal pancreas). Distended capillaries are distinctly visible in all parts of the section and their cellular contents can be plainly seen. The parenchyma as a whole seems quite well preserved, though there are areas aside from the islands of Langerhans which show well marked cloudy swelling. The islands of Langerhans with their irregular columns of polygonal cells and wide anastomosing capillaries, so aptly described by Osler, can be easily made out, their basement membrane is thickened and they present extensive changes; many of them show an extreme grade of hyaline degeneration with the cells completely disintegrated and their nuclei arranged in irregular groups crowded away from the capillaries, which are surrounded by a dense homogeneous layer of hyaline, staining with eosin a pale red. Around the edge of the island can be occasionally seen a connective tissue nucleus, giving evidence of the beginning inroad of scare tissue. The degree of degeneration is variable; some islands are completely replaced by hyaline and fail to show any semblance of cellular structure, and, while cloudy swelling is to be seen in its various stages in the majority of the islands, there are few with apparently normal cells presenting little or no change.

DIAGNOSIS.

Diabetes mellitus.

## Figures and Tables

**No. 1. f1:**
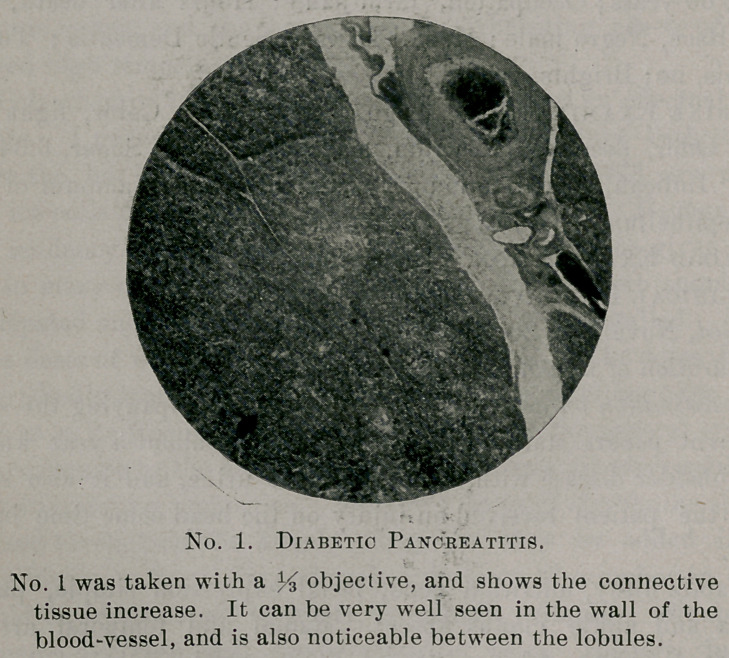


**No. 2. f2:**
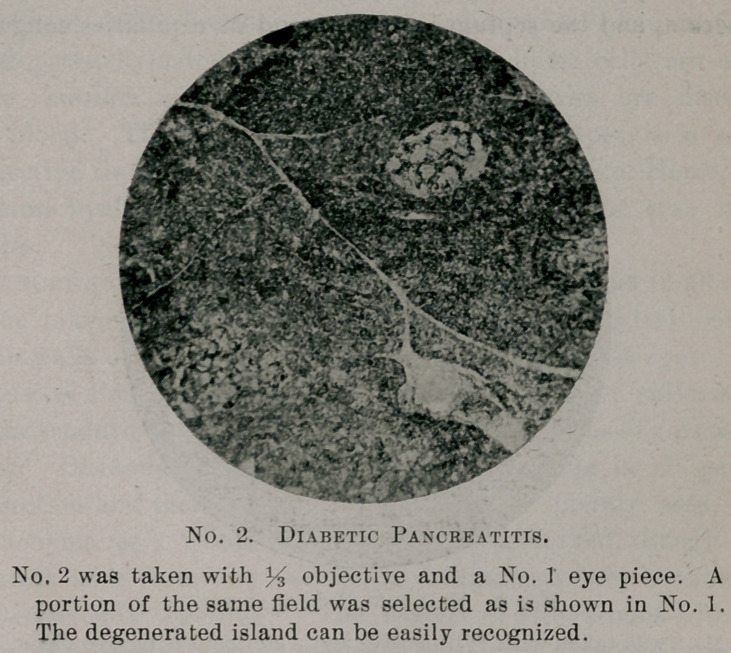


**No. 3. f3:**